# Maximum Entropy Modeling to Predict the Impact of Climate Change on Pine Wilt Disease in China

**DOI:** 10.3389/fpls.2021.652500

**Published:** 2021-04-23

**Authors:** Xinggang Tang, Yingdan Yuan, Xiangming Li, Jinchi Zhang

**Affiliations:** ^1^Co-Innovation Center for Sustainable Forestry in Southern China, Jiangsu Province Key Laboratory of Soil and Water Conservation and Ecological Restoration, Nanjing Forestry University, Nanjing, China; ^2^College of Materials Sciences and Technology, Guangdong University of Petrochemical Technology, Maoming, China

**Keywords:** climate change, pine wilt disease, species distribution model, risk prediction, pine species

## Abstract

Pine wilt disease is a devastating forest disease caused by the pinewood nematode *Bursaphelenchus xylophilus*, which has been listed as the object of quarantine in China. Climate change influences species and may exacerbate the risk of forest diseases, such as the pine wilt disease. The maximum entropy (MaxEnt) model was used in this study to identify the current and potential distribution and habitat suitability of three pine species and *B. xylophilus* in China. Further, the potential distribution was modeled using the current (1970–2000) and the projected (2050 and 2070) climate data based on two representative concentration pathways (RCP 2.6 and RCP 8.5), and fairly robust prediction results were obtained. Our model identified that the area south of the Yangtze River in China was the most severely affected place by pine wilt disease, and the eastern foothills of the Tibetan Plateau acted as a geographical barrier to pest distribution. Bioclimatic variables related to temperature influenced pine trees’ distribution, while those related to precipitation affected *B. xylophilus*’s distribution. In the future, the suitable area of *B. xylophilus* will continue to increase; the shifts in the center of gravity of the suitable habitats of the three pine species and *B. xylophilus* will be different under climate change. The area ideal for pine trees will migrate slightly northward under RCP 8.5. The pine species will continue to face *B. xylophilus* threat in 2050 and 2070 under the two distinct climate change scenarios. Therefore, we should plan appropriate measures to prevent its expansion. Predicting the distribution of pine species and the impact of climate change on forest diseases is critical for controlling the pests according to local conditions. Thus, the MaxEnt model proposed in this study can be potentially used to forecast the species distribution and disease risks and provide guidance for the timely prevention and management of *B. xylophilus*.

## Introduction

Pines are coniferous trees of the *Pinus* genus with high economic importance in China. They have broad application prospects in the lumber, fuel, and chemical industries (Karchesy, 1979; Thomas et al., 2007) and play important roles in soil and water conservation (Hongyan et al., 2003; Li et al., 2011b). Pine wilt disease, caused by the pinewood nematode *Bursaphelenchus xylophilus* (Steiner and Buhrer) Nickle, is one of the most devastating coniferous forest diseases (Mamiya, 1983) and a significant challenge to the pine industry. It invades other countries through interregional trade, causing catastrophic damage to the forest ecosystem. At present, more than 40 countries have listed it as a quarantine pest (Tóth, 2011). Among them, China is the most threatened country (Li et al., 2011a). Pine wilt disease was first discovered in China in 1982 in *Pinus thunbergii* of Nanjing. By 1999, the number of diseased and dead trees reached 5.5 million (Shen and Cao, 1995; Zhixin, 1995; Guo et al., 2001). So far, *B. xylophilus* has caused pine trees’ large-scale death in more than 10 provinces of China (Han et al., 2007). Although *P. thunbergii* and *Pinus densiflora* were the susceptible species initially, *Pinus massoniana*, a native tree species in China, has gradually become susceptible (Zhao, 2008). Studies have shown that areas with an average annual temperature of 10–14°C, especially the southern parts of Yellow River in China, are the likely distribution areas of pine wilt disease, indicating a threat from pine wilt disease to nearly 6,000 km^2^ of pine forests (Mamiya, 1983; Braasch, 2001; Diekmann, 2002).

Climate is one of the critical factors influencing vegetation’s type and distribution globally (Rutherford and Webster, 1987). The rise in carbon dioxide levels has increased air temperature and affected the natural ecosystem, including the correlation between plants and pests. The warming effect has also influenced the incidence and severity of forest diseases, and many insect pests have grown faster in response to the shortened growth cycle of plants (Trumble and Butler, 2009). Species distribution modeling of 30 prospective insect pest species (Coleoptera and Lepidoptera) in the Swedish boreal forest predicted a large increase in the distribution of these pests in the future, resulting in outbreaks in “new” areas (Hof and Svahlin, 2016). Besides, global warming may accelerate the spread of pine needle gall midge in the future, leading to an early outbreak in the plant growing season. Generally, high temperature induces stress in trees and exacerbates pest outbreaks and trees’ death (Raffa et al., 2015). However, plants and insect pests respond differently to climate change, which shows their particularity and differences in adapting to the environment. The species’ specific responses to climate change also influence species and climate interactions and exacerbate the uncertainties. A climatic anomaly of 2003 resulted in a rapid expansion of the pine processionary moth (Battisti et al., 2006). Climate change can also have a multifaceted impact on the distribution and reproduction of pest species such as *Thaumetopoea pityocampa* (Hódar et al., 2003). These findings indicate a significant effect of climate change on plants and disease incidence. Therefore, it is urgent to predict the trends in plants and diseases under climate change.

The niche model can be used to assess and predict the effect of climate change on plants and pest insects. Currently, several models, such as bioclimatic modeling (BIOCLIM), global geographic information system for a medicinal plant (GMPGIS), climate change experiment (CLIMEX), genetic algorithm for rule-set production (GARP), and maximum entropy (MaxEnt; Beaumont et al., 2005; Pattison and Mack, 2008; Du et al., 2017; Zhang et al., 2018b), have been used to predict the potential distribution of species. Among these, the MaxEnt-based model MaxEnt is a frequently used tool. The theory of maximum entropy was first proposed in 1957 (Phillips et al., 2006). The Java MaxEnt model, which was developed based on this theory has become the most commonly used species distribution model (SDM; Phillips et al., 2006). It can assess the potential distribution of diseases and insect pests and reasonably predict areas where disease symptoms may occur under climate change conditions (Yu et al., 2012). The MaxEnt model has been used in research on natural reserve design, endangered species survey, alien species risk assessment, and climate change impact (Khanum et al., 2013; Padalia et al., 2014; Liu et al., 2015). The MaxEnt model predicted the potential geographic distribution of *Phenacoccus solenopsis* in India, which helps in pest and disease management (Fand et al., 2014). The model also predicted the potential distribution of the pest *Euplatypus parallelus* and recommended strengthening the quarantine measures in the tropical and subtropical regions of southern China (Tang et al., 2019). MaxEnt is a useful and accurate prediction model, which has the advantages of supporting multiple variables, small sample requirements, high flexibility, and easy result interpretation (Xiong et al., 2004; Yuan et al., 2015). It can accurately predict the suitable habitats for endangered species, even for small samples (Kumar and Stohlgren, 2009).

In this study, we used a MaxEnt model based on the distribution area of three pine species and *B. xylophilus* to explore and predict the risk of pine wilt disease in 2050 and 2070 under two distinct climate change scenarios. The occurrence records of three pine species were collected using databases [Global Biodiversity Information Facility (GBIF), China Virtual Herbarium (CVH), and China’s eFlora website] and literature search.^1^ Based on the environmental factors suitable for pine trees’ growth and pine wilt disease, MaxEnt and ArcGIS platform were used for modeling and analysis. We aimed to (1) identify the critical environmental factors affecting the geographical distribution of three pine species and *B. xylophilus* based on climate similarity and combining the environmental factors and occurrence records to establish MaxEnt models; (2) predict the areas suitable as habitats of pine species and *B. xylophilus* under two representative concentration paths (RCP 2.6 and 8.5) at different periods (current, 1950–2000 and future, 2050–2070) based on MaxEnt and ArcGIS; (3) systematically analyze the present situation and the spatiotemporal changes in pine species and *B. xylophilus* caused by climate change; and (4) and expound the impact of global warming and propose future research directions. Our study’s findings will provide a theoretical basis for introducing and cultivating pine trees to achieve sustainable forest development.

## Materials and Methods

### Species Selection and Occurrence Records

**Figure 1:**
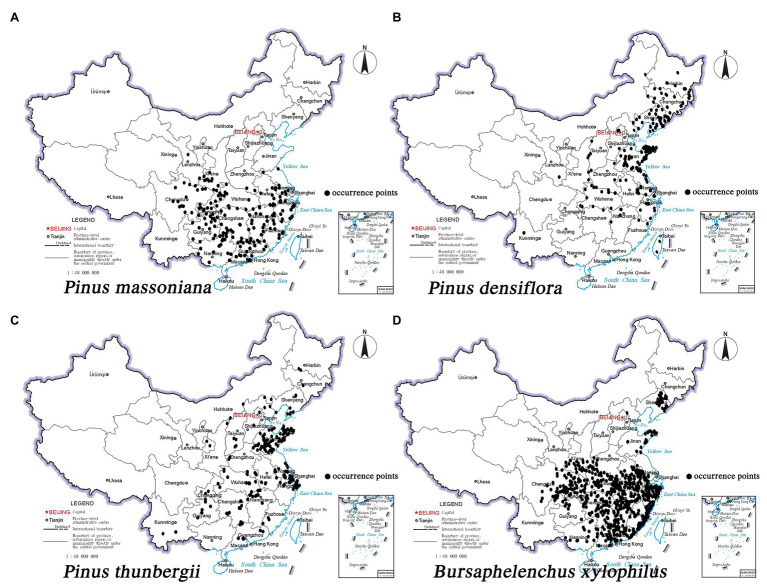
Occurrence records of three pine species and *Bursaphelenchus xylophilus* in China. **(A)** *Pinus massoniana*; **(B)** *Pinus densiflora*; **(C)** *Pinus thunbergii*; **(D)** *B. xylophilus*.

The primary susceptible hosts of *B. xylophilus* in China are *P. densiflora* and *P. thunbergii*. The native tree species *P. massoniana* also has gradually become sensitive to *B. xylophilus*. Meanwhile, pine wilt disease has mostly affected *P. massoniana*, *P. densiflora*, and *P. thunbergii* in Japan, South Korea, and China. Therefore, we selected these three representative pine species for the study. The sampling points of the three pine species and *B. xylophilus* are shown in Figure 1. The occurrence records of the pine species were derived from the online databases GBIF,^2^ CVH,^3^ China’s eFlora website,^4^ and China National Knowledge Infrastructure (CNKI).^5^ Data on the epidemic areas of pine wilt disease were obtained from the National Forestry and Grassland Administration (NFGA).^6^ The data points were cleaned by removing the duplicates. Specimen details with unknown geographic coordinates also were removed, and the plant catalog of the sample plots was verified. For the available records lacking coordinates, GoogleEarth was used to query the latitude and longitude information corresponding to the place names. Information on the distribution of *P. massoniana* (178 points), *P. densiflora* (139 points), *P. thunbergii* (156 points), and *B. xylophilus* (473 points) was obtained and saved as a “.csv” file for later use. Finally, ArcGIS 10.5 mapping software (Environmental Systems Research Institute, ESRI, Redlands, California, United States) was used with a 1:400,000 scale vector map of China’s administrative divisions and “.csv” files of the three pine species and *B. xylophilus* to map the areas suitable as habitat for the species. ArcGIS 10.5 is a comprehensive geographic information system developed by ESRI and used for layer and data format conversion and reclassification. The national basic geographic information system supplied the vector map of China’s administrative divisions.^7^ ArcGIS 10.5 was used to determine the center of gravity of the suitable habitats for the three pine species at different periods to judge their migration status in response to climate change.

### Environmental Variables

In this study, 19 environmental variables were selected to study the correlation between bioclimatic variables and the distribution of the three pine species and *B. xylophilus*. The bioclimatic variables version 1.4 were downloaded from the WorldClim dataset,^8^ and the coordinate system was WGS84 (Hijmans et al., 2005). The bioclimatic variables of future climate included two representative concentration paths (RCP 2.6 and 8.5). The RCP2.6 and RCP8.5 selected in this study represented low and high greenhouse gas emission scenarios in the future, respectively. These are the most recent global climate models climate projections that are used in the Fifth Assessment IPCC report. These bioclimatic variables are listed in Table 1.

**Table 1:** Bioclimatic variables used to predict the current and future distribution of three pine species and *B. xylophilus* in China.

Type	Variables	Description	Units
Bioclimatic variables	Bio1	Annual mean temperature	°C
	Bio2	Mean diurnal range [Mean of monthly (max temp - min temp)] (°C)	°C
	Bio3	Isothermality (Bio2/Bio7) (×100)	-
	Bio4	Temperature seasonality (SD×100; Coefficient of variation)	°C
	Bio5	Max temperature of warmest month	°C
	Bio6	Min temperature of coldest month	°C
	Bio7	Temperature annual range (Bio5-Bio6)	°C
	Bio8	Mean temperature of wettest quarter	°C
	Bio9	Mean temperature of driest quarter	°C
	Bio10	Mean temperature of warmest quarter	°C
	Bio11	Mean temperature of coldest quarter	°C
	Bio12	Annual precipitation	mm
	Bio13	Precipitation of wettest month	mm
	Bio14	Precipitation of driest month	mm
	Bio15	Precipitation seasonality (Coefficient of variation)	-
	Bio16	Precipitation of wettest quarter	mm
	Bio17	Precipitation of driest quarter	mm
	Bio18	Precipitation of warmest quarter	mm
	Bio19	Precipitation of coldest quarter	mm

Multicollinearity among environmental variables can lead to overfitting of the model prediction results (Graham, 2003). To avoid overfitting and select the most fitting variables, this study used Pearson’s correlation coefficient (r) method to test the correlation among the 19 environmental variables. Pearson’s correlation coefficient greater than 0.8 indicates a strong correlation between the two variables. One of the two related variables was removed to ensure the model simulation accuracy (Zhang et al., 2016; Yan et al., 2020). This study, combined with the relevant earlier studies, selected nine, six, nine, and nine environmental variables, respectively, for the subsequent analysis of *P. massoniana*, *P. densiflora*, *P. thunbergii*, and *B. xylophilus*. The key environmental factors used to establish the potential distribution models of pine species and *B. xylophilus* are listed in Table 2.

**Table 2:** Major bioclimatic variables affecting the habitat distribution of three pine species and *B. xylophilus* in China.

Species	Environmental variables
*Pinus massoniana*	Bio1, Bio2, Bio3, Bio4, Bio8, Bio12, Bio14, Bio15, Bio18
*Pinus densiflora*	Bio1, Bio2, Bio3, Bio12, Bio13, Bio15
*Pinus thunbergii*	Bio1, Bio2, Bio3, Bio4, Bio5, Bio8, Bio12, Bio13, Bio15
*Bursaphelenchus xylophilus*	Bio1, Bio2, bio3, Bio5, Bio8, Bio12, Bio14, Bio15, Bio18

### MaxEnt Modeling

The MaxEnt software (version 3.4.1),^9^ based on sample point information of the species and the environment variables, was used for analysis and predict the distribution of suitable habitats of the species. The selected bioclimatic variables and occurrence data of pine species and *B. xylophilus* were uploaded to MaxEnt for modeling and predicting the distribution. Here, 75% of the occurrence records were used to train the model, and the remaining 25% were used to test the model’s predictive ability (Yan et al., 2020). The model ran 500 iterations with the default parameter settings, and the prediction output was saved in “.asc” format (Yan et al., 2020). Feature combination (FC) and regularization multiplier (RM) were optimized through R 3.6.1, and other calculation parameters were kept as the default.

Furthermore, the receiver operating characteristic (ROC) curve of the jackknife method was used to evaluate the model’s reliability (Dorfman et al., 1992). The area under the ROC curve (AUC) was used to assess the potential SDM (Li et al., 2020). AUC value, ranging from 0 to 1, is positively correlated with the model’s prediction accuracy; the higher the AUC value, the higher its reliability (Wiley et al., 2003). AUC value greater than 0.9 indicates SDM robustness (Howell et al., 2011; Buckman-Sewald et al., 2014). The artificial classification method with a better division effect was adopted to discriminate the areas based on ecological habitat suitability based on the existence probability of the pine species and the pine wilt disease. The potential species distribution map predicted by the MaxEnt model was in the logistic format. The predicted existence probabilities of the species between 0 and 1 were reclassified through the reclassification function of ArcGIS 10.5, and four categories of habitat suitability were defined as follows (Yan et al., 2020): high suitability (0.6–1), medium suitability (0.4–0.6), low suitability (0.2–0.4), and no suitability (0–0.2). Meanwhile, the jackknife test was used to generate a response map of environmental factors in MaxEnt. In this study, areas with an existence probability greater than 0.6 were considered the most suitable (Hemery et al., 2016).

## Results

### Evaluation of the Accuracy and Contribution of Variables

Predicting the current and future distribution of pine species and pine wilt disease can be used to estimate forest disease risks under climate change. In this study, AUC values greater than 0.9 indicated the prediction model’s high credibility and accuracy. The model performed well in matching the distribution of the occurrence records. The predicted current habitat suitability is consistent with the actual distribution of three pine species and *B. xylophilus*. Besides, the model predicted shifts in habitat suitability under future climate scenarios.

Furthermore, we examined the variable importance by the jackknife method. In this study, percentage contribution, a measure representing the importance and contribution of the variables to the prediction results, was used to distinguish the effect of different variables on the potential distribution area. Among the variables used in the *B. xylophilus* model, isothermality (Bio3), maximum temperature of warmest month (Bio5), annual precipitation (Bio12), and precipitation of driest month (Bio14) contributed the maximum; the total percentage contribution of these variables was more than 70%. Annual mean temperature (Bio1) and mean diurnal range (Bio2) were the two most important variables in predicting the probable distribution of *P. massoniana*, and the total percentage contribution of these two variables was above 65%. Isothermality (Bio3) and annual precipitation (Bio12) mainly affected the distribution of *P. densiflora*, and the total percentage contribution was above 60%. Meanwhile, for *P. thunbergii*, the total percentage contribution of mean diurnal range (Bio2), mean temperature of the wettest quarter (Bio8), and precipitation of the wettest month (Bio13) was above 45%.

### Current Risk of Forest Disease

**Figure 2:**
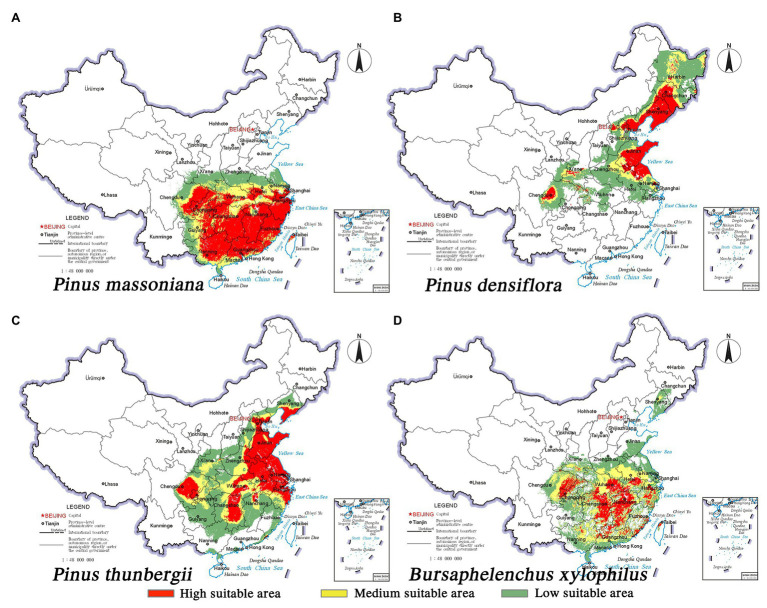
Habitat suitability maps showing the occurrence of three pine species and *B. xylophilus* in China under current climatic conditions. **(A)** *P. massoniana*; **(B)** *P. densiflora*; **(C)** *P. thunbergii*; **(D)** *B. xylophilus*.

**Figure 3:**
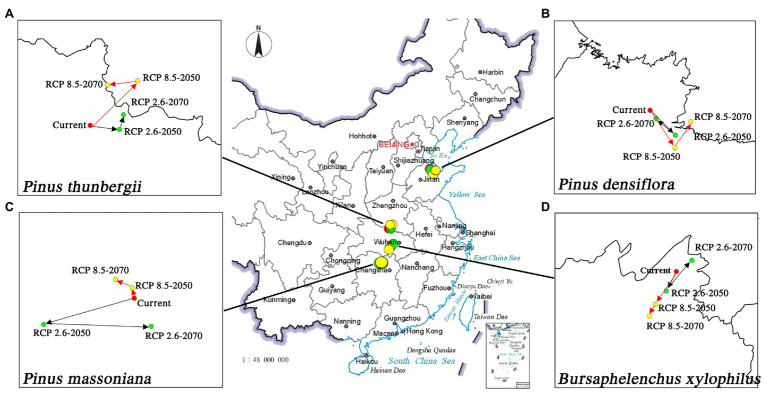
Migration of the geometric centers of suitable areas by 2050 and 2070 under two distinct climate change scenarios. **(A)** *P. thunbergii*; **(B)** *P. densiflora*; **(C)** *P. massoniana*; **(D)** *B. xylophilus*.

The predicted distribution of the three pine species and the insect pest is consistent with the current distribution. The nematode *B. xylophilus* showed the greatest overlap with the spatial distribution range of *P. massoniana*, followed by *P. thunbergii*; overlap was the least with *P. densiflora*. The potentially suitable habitats (high to low suitability) of *B. xylophilus* and *P. massoniana* were mainly distributed south of 34° north latitude. The Tibetan Plateau’s eastern foothills acted as a geographical barrier to pine species and pests (Figures 2A,D). It is worth noting that *B. xylophilus* was also distributed in the southern part of Shandong Province and the central and eastern parts of Liaoning Province. Meanwhile, the area suitable for *P. thunbergii* was distributed across the two major river basins of the Yangtze River and the Yellow River, with the highly suitable area mainly concentrated in the eastern coastal provinces, including Shandong, Liaoning, Jiangsu, Anhui, and Zhejiang (Figure 2C). The distribution range of *P. densiflora* was relatively narrow and long, and the highly suitable habitat was concentrated in Shandong and Liaoning provinces (Figure 2B). Compared with the other two pine species, *P. densiflora* was distributed in most parts of Heilongjiang Province. Unlike the center of gravity of the *P. densiflora* distribution area in Shandong Province, the center of gravity of the suitable habitats of *P. massoniana*, *P. thunbergii*, and *B. xylophilus* occurred on both sides of the Yangtze River, indicating big overlap in their habitats (Figure 3).

### Future Risk of Forest Disease

**Figure 4:**
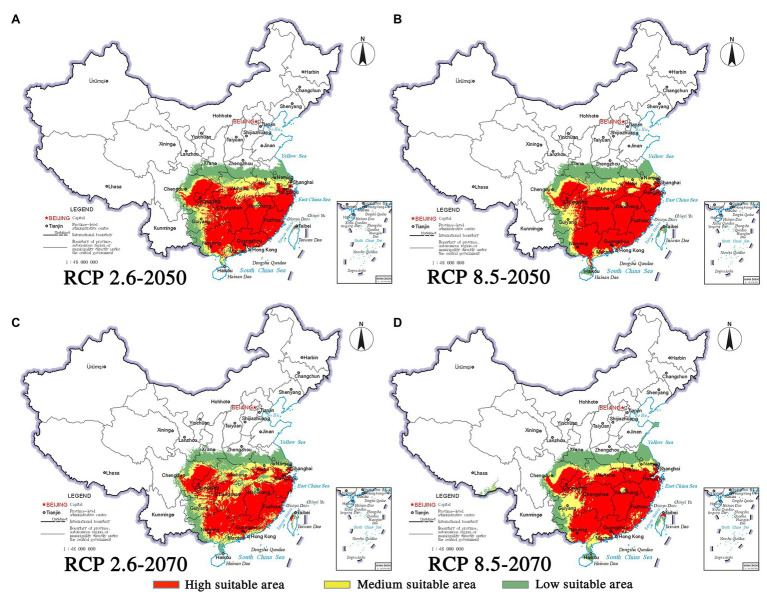
Habitat suitability maps showing the occurrence of *P. massoniana* by 2050 and 2070 under two distinct climate change scenarios in China. **(A)** RCP 2050-2.6; **(B)** RCP 2050-8.5; **(C)** RCP 2070-2.6; **(D)** RCP 2070-8.5.

**Figure 5:**
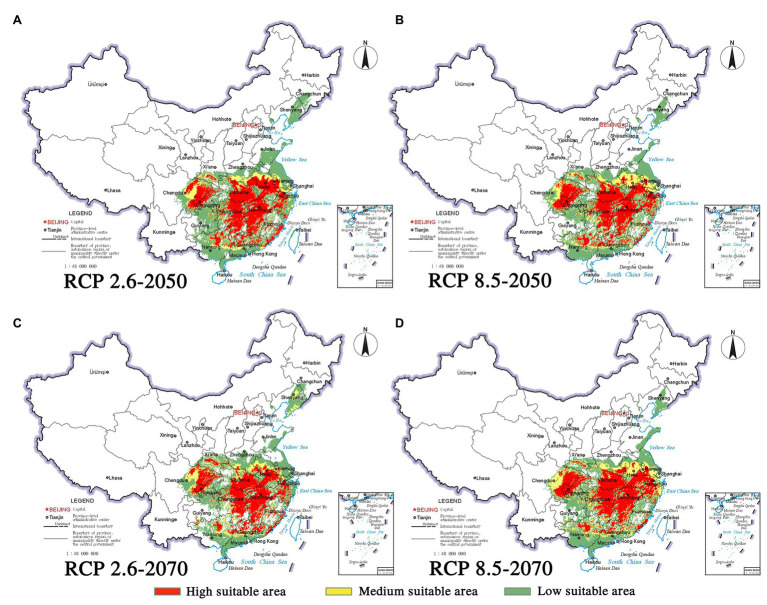
Habitat suitability maps showing the occurrence of *B. xylophilus* by 2050 and 2070 under two distinct climate change scenarios in China. **(A)** RCP 2050-2.6; **(B)** RCP 2050-8.5; **(C)** RCP 2070-2.6; **(D)** RCP 2070-8.5.

**Figure 6:**
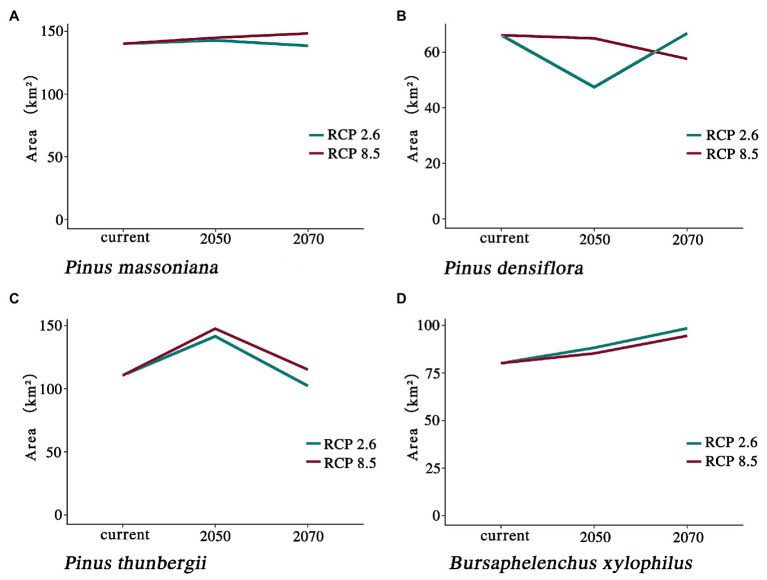
Changes in potentially suitable habitats by 2050 and 2070 under two distinct climate change scenarios. **(A)** *P. massoniana*; **(B)** *P. densiflora*; **(C)** *P. thunbergii*; **(D)** *B. xylophilus*.

**Figure 7:**
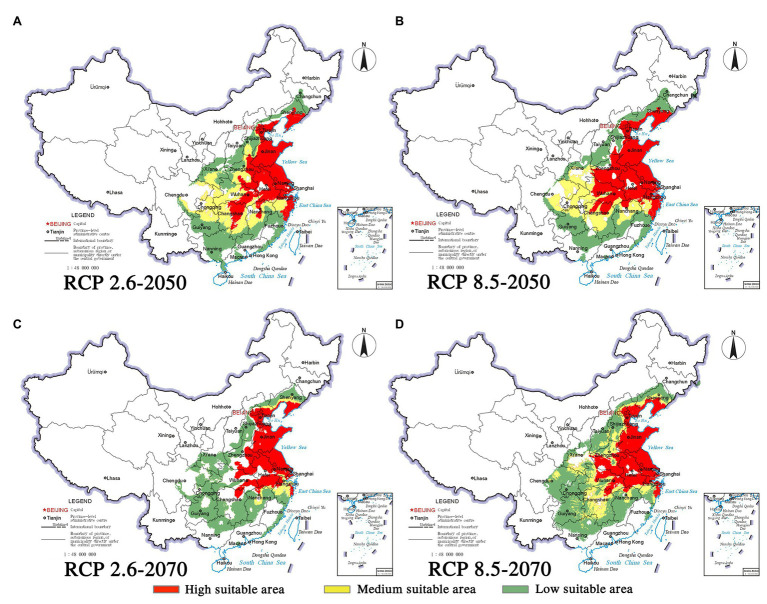
Habitat suitability maps showing the occurrence of *P. thunbergii* by 2050 and 2070 under two distinct climate change scenarios in China. **(A)** RCP 2050-2.6; **(B)** RCP 2050-8.5; **(C)** RCP 2070-2.6; **(D)** RCP 2070-8.5.

**Figure 8:**
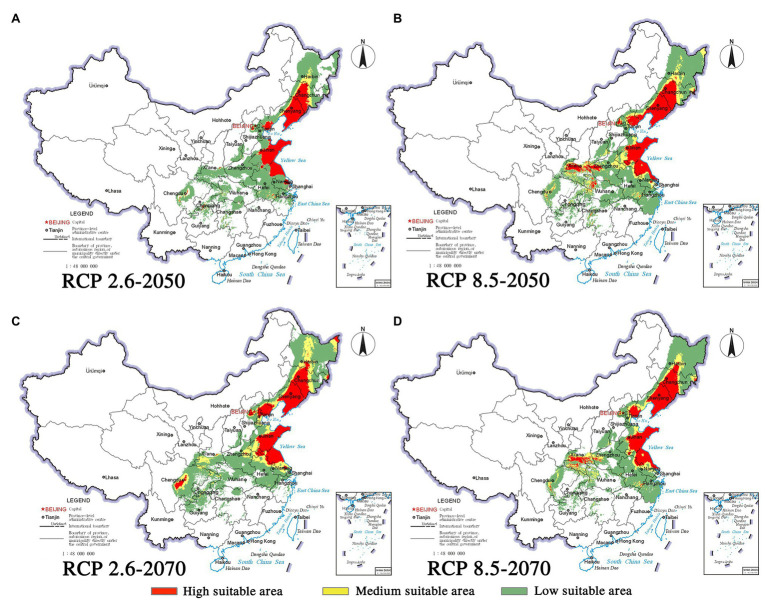
Habitat suitability maps showing the occurrence of *P. densiflora* by 2050 and 2070 under two distinct climate change scenarios in China. **(A)** RCP 2050-2.6; **(B)** RCP 2050-8.5; **(C)** RCP 2070-2.6; **(D)** RCP 2070–8.5.

Climate change will increase or reduce the risk of forest diseases to a certain extent by affecting the distribution of pine species and *B. xylophilus*. In the future, the spatial distribution range of *B. xylophilus* and *P. massoniana* will remain relatively stable (Figures 4, 5). Climate change will result in a continuous increase in the distribution area of *B. xylophilus* (Figure 6D), and the hazard area will be more extensive. Scenario RCP 8.5 indicated a continuous increase in the distribution area of *P. massoniana*, while scenario RCP 2.6 indicated an initial increase and then a decrease (Figure 6A). Meanwhile, *P. thunbergii* will expand to the southwest of China by 2050, and the distribution area will continue to increase (Figures 6C, 7). After 2050, the distribution area of *P. thunbergii* will show a declining trend. Under temperature rise in the future, the distribution range of *P. densiflora* will continue to shrink, and the distribution area will decrease (Figure 8). After 2050, the area will increase to a certain extent only under RCP 2.6 (Figure 6B).

For pine species and *B. xylophilus*, the center of gravity of suitable habitats will shift in response to future climate change. Under the conditions of RCP 2.6, the center of gravity of *B. xylophilus* will move back and forth in the southwest direction and then move to the northeast direction (Figure 3D). The center of gravity of *P. massoniana* will shift in the same direction as that of *B. xylophilus*. In contrast, the center of gravity of *P. thunbergii* and *P. densiflora* will move opposite to that of *B. xylophilus*. Under the RCP 8.5 scenario, the center of gravity of *B. xylophilus* will shift toward the southwest. However, the shift in the center of gravity of the three pine species is not consistent with that of *B. xylophilus* (Figure 3).

## Discussion

### Performance of the Species Distribution Modeling Approach

Pests have spread rapidly over the decades, and *B. xylophilus* has become one of the most important forest pests in Japan, China, South Korea, and Portugal (Evans et al., 1996; Smith et al., 1998; Sousa et al., 2011b; Tóth, 2011). Pine wilt disease has eventually spread to South Korea, accompanied by the death of many pine trees, which led to significant production losses (Mamiya, 1988; Ryss et al., 2011). Therefore, understanding the current distribution and predicting its future distribution under climate change is vital for future management. This study is the first to investigate the environmental characteristics of three different pine species and *B. xylophilus* in China using the MaxEnt model, to assess the current suitable areas of pine wilt disease, to determine the impact of climate change on this pest, and eventually to predict the risk of the forest disease. MaxEnt is a model widely used to study the suitable distribution areas of different species and has demonstrated good prediction accuracy (Kumar and Stohlgren, 2009; Khanum et al., 2013; Remya et al., 2015). Scientists have used the model to precisely assess the impact of climate change on plant distribution in recent years (Santana et al., 2019; Zhang et al., 2019). Besides, the validation statistic’s consistency for the pest and host plant projections in this study demonstrates the model’s robustness and reliability in predicting the highly suitable areas for pest development. However, the climatic variables are not the only determinants of habitat suitability. Therefore, the actual habitats of pine trees and *B. xylophilus* in China may be smaller than what is indicated by our model (Hirzel and Le Lay, 2008). Natural enemies, soil constraints, and anthropogenic activities will also affect the species’ spatial distribution under climate change (Wan et al., 2019). Studies based on the MaxEnt model have not addressed soil and human factors’ effects on future plant distribution (Kumar, 2012; Khanum et al., 2013; Zhang et al., 2018a) because current soil science research focuses only on a single soil component or a small geographic range, and the knowledge on future long-term soil changes is less (Walter et al., 2003; Schillaci et al., 2017). Moreover, it is difficult to simulate the future climate and soil trends under anthropogenic activities’ influence (Zhou et al., 2003). However, since the climate is the most important determinant of species distribution and invasion and provides necessary information on habitat suitability, we believe our model has provided a sufficiently accurate estimation and prediction (Broennimann et al., 2007; Gallardo and Aldridge, 2013). Besides, the AUC values greater than 0.9 indicated robust model performance.

### Climatic Space for Pine Species and *B. xylophilus*

Environmental factors affect the growth and development, geographical distribution, diversity, and richness of species (Austin, 2002; Kreft and Jetz, 2007). Therefore, future climate change will inevitably affect the distribution of pine species and *B. xylophilus*. These changes will bring new challenges to the control and management of forest diseases. Analysis based on the MaxEnt model identified annual mean temperature (Bio1), mean diurnal range (Bio2), isothermality (Bio3), annual precipitation (Bio12), and precipitation seasonality (Bio15) as the common climate factors affecting *B. xylophilus* and pine species. The similarity in niche requirements provides substantial ecological evidence for continuous *B. xylophilus* damage in pine trees (Peterson and Soberón, 2012). In this study, the environmental factors affecting *P. massoniana* and *P. thunbergii* were similar to those affecting *B. xylophilus*. In contrast, the factors affecting *P. densiflora* and *B. xylophilus* were relatively different. This observation agrees with the evidence that environmental variables affecting species’ distribution often have a significant correlation with their geographical distribution (Lee and Mitchell-Olds, 2011). Relevant records and model simulations have proven that *P. massoniana* and *P. thunbergii* are mainly distributed in the south of 40°N latitude in China, and *P. thunbergii* is primarily distributed in the vast area south of the Yangtze River (Businský, 2008). Our observations and earlier reports together indicate that the higher the overlap of geographical distribution, the more similar the environmental factors. Consequently, this resulted in more significant damage by *B. xylophilus* in *P. massoniana* and *P. thunbergii* than *P. densiflora* (Kim et al., 2020).

Among the factors affecting pine species, the temperature-related factors demonstrated a greater contribution rate, which indicates higher sensitivity of pine species to temperature changes. However, different pine species have different sensitivity to temperature; ponderosa pine seedlings were more sensitive to temperature fluctuations than lodgepole pine seedlings (Petrie et al., 2016). Meanwhile, the bioclimatic variables related to precipitation demonstrated significant roles in the spatial distribution projections of *B. xylophilus* in the proposed model. Drought significantly increased the population density and damage of *B. xylophilus* (Han et al., 2003). A previous study (Kanzaki and Giblin-Davis, 2018) also found that during the years when *B. xylophilus* damage was severe, the areas affected were mainly under high temperature and drought conditions. Additionally, *B. xylophilus* outbreak is negatively correlated with the stem water content (Woo et al., 2009). Sousa et al. (2011a) studied the importance of the moisture content in the wood for pine wood nematode survival, and confirmed that nematode survival and movement increase with lower moisture content. Besides, the stem and leaf’s relative water content gradually reduced in the pine tree inoculated with *B. xylophilus* before the symptoms appeared (Tan et al., 2005). *Hyphantria cunea* is another important pest of China, and unlike *B. xylophilus*, *H. cunea*’s invasion and reproduction were strongly affected by temperature. Deviation from normal growth temperature affected gonad development and energy metabolism and led to abnormal reproduction (Janowitz and Fischer, 2011). The low temperature in winter limited the occurrence and spread of *H. cunea*, while the longer growing season and higher effective temperature sum (ETS) promoted the overwintering of pests (Carrlee, 2003; Hakala et al., 2011). Less rainfall and low average annual temperature in northern China have increased the demand of *H. cunea* for temperature. The difference in the two main forest pests’ niche requirements may be due to their geographical distribution differences. The pest *B. xylophilus* needs a relatively dry environment. Based on the climatic conditions, we should pay special attention to the occurrence of *B. xylophilus* in a drought year. Pest monitoring and forecasting will help in timely management.

### Suitable Habitat and Its Dynamics

The climate change impact is reflected in the distribution of plants and animals (Lewis, 2006). In this study, MaxEnt and ArcGIS were used to understand the current distribution and predict the future distribution under climate change in China. The MaxEnt model revealed that the shift in the center of gravity of the distribution areas was significantly different under the representative concentration paths RCP 2.6 and RCP 8.5. Under RCP 2.6 condition, the shift in the center of gravity of *P. massoniana* distribution area was similar to that of *B. xylophilus*. Similar niche demands indicate their consistent response to low representative concentration pathways (RCP 2.6; Juan et al., 2008; Gao et al., 2017). The similar distribution range and shift in the center of gravity of *P. massoniana* and *B. xylophilus* indicate that *P. massoniana* will face a continuous disease risk under this scenario. Meanwhile, the three pine species migrated opposite to *B. xylophilus* under RCP 8.5 condition; the pine trees showed an evident northward migration. Similarly, the improved BIOME4 model used to simulate natural vegetation distribution in China predicted a northward movement of the vegetation boundary in eastern China under future climate warming (Zhao and Wu, 2014). However, the needle-shaped pine leaves can effectively prevent the evaporation of water and reduce the sensitivity to temperature changes, and therefore, the distance of migration will be relatively short (Dong-Mei et al., 2009). The short distance migration of pine species and the significant overlap with *B. xylophilus* distribution indicate the severity of forest diseases from another aspect will increase in the future. Due to global warming, precipitation in most parts of the northern hemisphere has increased significantly (Guo et al., 2019). In contrast, research predicts an arid future for China (Simpson et al., 2019). The drought in China in the future will promote the distribution range of *B. xylophilus*. The center of gravity of the suitable area for *B. xylophilus* will continuously move to the south. The suitable area will expand, especially under the condition of a high representative concentration pathway (RCP 8.5). On the contrary, Xu et al. (2015) predicted that under the same climate change conditions, the suitability of *H. cunea* at the junction of the four provinces of the North China Plain and in the United States might continue to weaken. Higher ambient temperature and humidity are conducive to the survival and reproduction of *H. cunea*, while *B. xylophilus* is more adaptable to arid climate conditions. Meanwhile, Yoshimura et al. (1999) suggested an influence of *Monochamus alternatus* on the distribution dynamics and damage degree of the suitable area of *B. xylophilus*. Their investigation of the correlation between temperature and *B. xylophilus* revealed that low temperature may inhibit the spread of *B. xylophilus* by affecting the reproduction and activity range of *M. alternatus*. Therefore, global warming is conducive to the activity of the nematode vector *B. xylophilus*, which will significantly promote the damage by pine wilt disease (Jikumaru and Togashi, 2000). The prevention and control of *B. xylophilus* in China’s southern parts must be strengthened to protect large-scale pine trees from pest invasion in the future. Besides, it is interesting that different pine species will respond differently under the various climate change scenarios due to the differences in their geographical distribution. Our study indicates that, compared with the relatively stable suitable areas of *P. massoniana*, the suitable areas of *P. densiflora* and *P. thunbergii* will fluctuate significantly under climate change. Zhengang et al. (2019) predicted a substantial expansion in the suitable area of *Larix principisrupprechtii* and a significant reduction in that of *Pinus tabulaeformis* under climate change; meanwhile, the suitable area of *Quercus mongolica* will remain relatively stable (Zhengang et al., 2019). The differences of suitable area change reported in the present and earlier studies suggest that the different species will respond differently to external changes. The specificity is closely related to each species’ climate affinity (Gómez-Mendoza and Arriaga, 2007). Environmental factors affect the natural spread of pests and further threaten the forest ecosystem (Siegert et al., 2015). However, various factors that may affect the species distribution, such as geographic barriers, human activities, and natural enemies, have not been considered in predicting the dynamic changes in suitable areas based on the model (Radosavljevic and Anderson, 2014). Besides, the evolution and adaptation of *B. xylophilus* have not been considered in the modeling process (Haines et al., 2009). Thus, the model, with certain limitations, can be used to predict forest diseases such as the pine wilt disease, and this prediction based on occurrence records and bioclimatic variables will help prevent the disease and nematode attacks (Wang et al., 2010; Wei et al., 2020).

## Conclusion

Species distribution modeling is an easy-to-implement predictive tool based on machine learning that is useful in the prediction of climate-affected species distribution changes. We assessed the correlation between environmental variables and the research objects, and found that the bioclimatic variables related to temperature affect and limit the distribution of pine trees, while those related to precipitation affect the distribution of *B. xylophilus*. Furthermore, MaxEnt model predicted the current and future distribution of three pine species and *B. xylophilus* and evaluated the risk of *B. xylophilus* attack in 2050 and 2070 under different climate change scenarios. The expected precipitation reduction in China under climate change will promote the spread of *B. xylophilus* and increase the pressure on disease prevention and control. We also found that *P. massoniana* and *B. xylophilus* responded similarly to climate change under RCP 2.6, which indicates that *P. massoniana* will continue to face disease risks under this scenario. Meanwhile, the shift in the center of gravity of the three pine species will be inconsistent with *B. xylophilus* under RCP 8.5, and the suitable areas of pine trees will move northward, which may reduce the risk of disease to a certain extent. But the suitable area of *B. xylophilus* will continue to expand under climate change, especially after 2070, which indicates that the control of forest pests will still be challenging. Overall, our work proposes a model based on significant bioclimatic variables to predict the impact of climate change on disease risks, which will help plan management strategies. Furthermore, the influence of natural enemies, soil and human activities on the distribution of species suitable habitats under climate change will be an important research direction in the future.

## Data Availability Statement

The original contributions presented in the study are included in the article/supplementary material; further inquiries can be directed to the corresponding author.

## Author Contributions

YY and XT: conception and design of the research. XT: acquisition of data. YY: analysis and interpretation of data. XT and XL: statistical analysis. YY and JZ: drafting the manuscript. All authors contributed to the article and approved the submitted version.

### Conflict of Interest

The authors declare that the research was conducted in the absence of any commercial or financial relationships that could be construed as a potential conflict of interest.

## Footnotes

^1^https://www.cnki.net/

^2^http://www.gbif.org/

^3^http://www.cvh.ac.cn/

^4^http://www.efloras.org/

^5^http://www.cnki.net/

^6^http://www.forestry.gov.cn/main/index.html

^7^http://bzdt.ch.mnr.gov.cn/index.jsp

^8^http://www.worldclim.org/

^9^http://biodiversityinformatics.amnh.org/open_source/maxent/
